# Asymptomatic Tuberculosis in the Namibia National Tuberculosis Prevalence Survey: Occurrence Under Various Case Definitions

**DOI:** 10.1111/tmi.70206

**Published:** 2026-07-28

**Authors:** Nunurai Ruswa, Gunar Gunther, Farai Mavhunga, Frank Cobelens, Mareli Claassens

**Affiliations:** ^1^ School of Medicine University of Namibia Windhoek Namibia; ^2^ National Tuberculosis and Leprosy Programme Ministry of Health and Social Services Windhoek Namibia; ^3^ Department of Pulmonology, Allergology and Clinical Immunology Inselspital, Bern University Hospital, University of Bern Bern Switzerland; ^4^ World Health Organization Geneva Switzerland; ^5^ Amsterdam University Medical Centres University of Amsterdam Amsterdam the Netherlands; ^6^ Desmond Tutu TB Centre Stellenbosch University Stellenbosch South Africa

**Keywords:** asymptomatic, screening, subclinical, TB, tuberculosis

## Abstract

**Background:**

Asymptomatic tuberculosis (TB) is emerging as a disease state warranting attention because it is presumed infectious, yet affected individuals lack symptoms. The World Health Organization recommends screening for cough, fever, weight loss and night sweats and recently defined asymptomatic TB. However, the place of these symptoms in the definition has not yet been standardised. We determined the proportion of patients with bacteriologically confirmed asymptomatic TB without four symptom definitions: persistent cough (> 2 weeks), cough of any duration, W4SS of any duration or W4SS for > 2 weeks.

**Methods:**

Secondary analysis of the Namibia TB prevalence survey data was performed; a representative sample had been interviewed for symptoms, underwent chest radiography and was offered bacteriological testing. The proportion of asymptomatic TB was computed for each definition. Logistic regression was used to determine factors associated with asymptomatic TB across four definitions.

**Results:**

Among 29,495 participants in the nationally representative sample (86.8% of the target sample size), 123 TB cases met the case definition. Mean age was 45 years, and 74 (60%) were male. Of TB cases, 60/123 (48.8%) reported a cough of any duration, 20/123 (16.3%) a fever of any duration, 25/123 (20.3%) weight loss and 26/123 (21.1%) night sweats. Asymptomatic TB ranged from 39.8% to 63.0% by definition, with screening based on prolonged cough missing 63.0% of bacteriologically confirmed TB patients, while screening based on W4SS any duration missed 39.8% of TB cases.

**Conclusion:**

Even when using an inclusive symptom screening system that includes any duration of the W4SS, asymptomatic TB occurred in 39.8%, highlighting a public health concern for asymptomatic TB. There is a need to standardise the symptoms being used when defining asymptomatic TB and we recommend the W4SS of any duration.

## 1 Introduction

Tuberculosis (TB) remains a leading cause of death among infectious diseases, killing an estimated 1.25 million people, more than any other infectious disease, in 2023 when only 75% of the estimated 10.8 million incident TB cases were notified and treated [1]. Although patients with TB are typically diagnosed and treated following presentation with recognisable symptoms, it is becoming increasingly apparent that many patients remain undiagnosed because they do not present with symptoms [2, 3]. Patients in this state, previously referred to as subclinical TB and recently redefined as asymptomatic TB [1, 4], often go undetected and may be a significant public health threat, excreting bacilli through breathing and possibly contributing to ongoing disease transmission despite the absence of cough [5, 6, 7, 8]. The concept of asymptomatic TB (aTB) has not been as widely discussed or investigated as the latent or active variants [9, 10]. A definition of subclinical TB was proposed by Drain et al. referring to disease due to viable *Mycobacterium tuberculosis (Mtb)* that “does not cause clinical TB‐related symptoms but causes other abnormalities that can be detected using existing radiologic or microbiologic assays” [11]. In early 2024, the International Consensus on Early TB States (ICE‐TB) came up with four distinct states of asymptomatic TB based on three dimensions: macroscopic pathology, infectiousness and the presence of symptoms [4].

Based on this consensus, the World Health Organization (WHO) [1] proposed a definition of aTB for the first time in its most recent 2024 Global TB Report. As defined by the WHO, aTB refers to a person with TB who does not report symptoms suggestive of TB during screening and may be bacteriologically confirmed or unconfirmed. According to the WHO, this definition aims to assist in the programmatic management of TB. Unfortunately, the WHO definition does not outline the suite of symptoms to be used in ensuring a consistent definition, presenting a challenge in determining the prevalence of aTB and comparing rates across programmes and countries given the variation in screening algorithms, a concern raised by some authors [12, 13, 14].

In many settings, symptom screening focuses on prolonged cough [14], whereas in people living with HIV, the WHO recommends including four symptoms (cough of any duration, fever, weight loss or night sweats), frequently referred to as W4SS [15]. The sensitivity of prolonged cough alone in detecting active TB is low, particularly in high burden HIV settings, despite its wide use. Addition of other symptom screening rules to the prolonged cough such as any three of six (cough < 2 weeks, night sweats, weight loss, fever, chest pain, shortness of breath) or any two of three (cough < 2 weeks, night sweats and weight loss) has been found to increase sensitivity while maintaining high specificity (> 84%) even in the absence of radiography [16]. In people without HIV, the success of the W4SS list has not been extensively evaluated and its use is likely to place a significant strain on resources if the outcome of symptom screening requires further investigation. Additionally, the prevalence of aTB has been best estimated from prevalence surveys as it is unlikely to present in the routine healthcare system. Although the rates of aTB from these estimates range from 36.9% to 79.7% of bacteriologically confirmed cases [2], no such analysis has been published in Namibia. In this study, we estimated the prevalence of aTB among bacteriologically confirmed TB cases from a nationwide prevalence survey and evaluated associated risk factors for aTB after applying four different suites of symptoms to define aTB.

## 2 Methods

### 2.1 Study Design and Setting

This study was a secondary analysis of data obtained from Namibia's first TB prevalence survey, a cross‐sectional study conducted between 2017 and 2018. At the time, the estimated population was 2.5 million inhabitants and this survey estimated the prevalence of bacteriologically confirmed TB to be 465 per 100,000 [17]. A survey TB case was defined as a participant that had bacteriological confirmation by mycobacterial culture or positive Xpert MTB/RIF and a TB‐consistent chest radiograph.

### 2.2 Definitions

We analysed the aTB prevalence according to the following four definitions: three definitions used in a recent meta‐analysis [18] plus an additional definition incorporating persistent W4SS. These four definitions of aTB were analysed in line with what has been reported in the literature [18, 19] and based on symptoms, as follows:

- aTB Definition 1: Survey case definition with no persistent cough for 2 weeks or more.
- aTB Definition 2: Survey case definition with no cough (any duration).
- aTB Definition 3: Survey case definition with no symptoms of any duration according to the WHO four‐symptom questionnaire (W4SS).
- aTB Definition 4: Survey case definition without any of the four symptoms of cough, fever, weight loss or night sweats (W4SS) lasting 2 weeks or more.

The first three definitions have been used in a recent meta‐analysis on asymptomatic TB [18], while the fourth is an adaptation of what was used in local guidelines [20].

### 2.3 Patient Selection, Sample Size and Sampling Strategy

The Namibian TB prevalence survey sample was the study population, with eligibility for this analysis based on having fulfilled the survey case definition. According to this definition, a survey TB case had bacteriological confirmation with mycobacterial culture or positive Xpert MTB/RIF (Cepheid, Sunnyvale, California) and a TB‐consistent chest radiograph. The primary study was conducted according to the WHO‐issued guidelines [21] and followed WHO recommendations for multilevel cluster sampling, stratified by region and setting with probability proportionate to size. A cluster was a geographic area also described locally as an “enumeration area”, of which 68 were randomly selected within the respective strata. Following house‐to‐house enumeration of all households within a cluster, eligible individuals were invited to participate in screening. Eligible participants were at least 15 years of age and had resided within the selected clusters for at least 2 weeks. TB screening entailed symptom interviews and chest radiography for all participants, followed by sputum testing for those who screened positive. Participants were evaluated for cough, fever, night sweats and weight loss as well as their respective durations. TB screening was considered positive if any of the W4SS were present or when any abnormality was detected on chest radiography as determined by a medical officer or a high computer aided detection score, threshold ≥ 60. Two sputum samples were tested using Xpert MTB/RIF (Cepheid, Sunnyvale, California) on the first sample and mycobacterial culture in the BACTEC Mycobacterial Growth Indicator Tube (MGIT) liquid media (Becton, Dickinson and Company, Franklin Lakes, New Jersey) on the second sample. A TB case was defined in this survey as a participant with two positive bacteriological results or one positive plus chest radiographic finding consistent with TB, as read by a central radiologist and agreed upon in a clinical panel.

### 2.4 Data Collection

During the survey, the data were entered into a customised MySQL‐based database with a PHP‐based interface (iMarketing, Windhoek, Namibia). All participants in the survey were offered symptom screening and a posteroanterior chest X‐ray using either a Digital Delft DI‐Canon EasyDR or Delft DI‐Canon EasyPortable X‐ray system (Delft Imaging Systems, Veenendaal, Netherlands). Additional radiographic image analysis was provided with Computer‐Aided Detection for Tuberculosis version 5 (CAD4TB; Delft Imaging Systems, Veenendaal, Netherlands), with scores being assigned from 1 (least likely to have TB radiologically) to 100 (most likely to have TB radiologically). Sputum positivity was defined as any positive result on Xpert MTB/RIF (Cepheid, Sunnyvale, California) or mycobacterial culture.

### 2.5 Data Management and Analysis

Participant unique identifiers, symptoms, bacteriological results, radiological results, age, sex, HIV status, height, weight, TB history, other medical history and other sociodemographic factors were among the variables analysed for the present study in Stata version 18 (Statacorp, College Station, Texas). We computed the proportion of TB cases with each of the four symptoms (cough, fever, weight loss and night sweats) in turn and presented the combination of the symptoms in an upset plot to better visualise the overlap. We accounted for the complex survey design (stratification by region and geography, clustered sampling and sampling weights) using Stata's *svy* commands. Survey‐weighted proportions for each definition of aTB and 95% confidence intervals were calculated with Taylor series linearisation.

In bivariate analysis, the chi‐square test was used to test the factors associated with aTB (each definition in turn) out of age‐group, sex, geography, HIV status, body mass index (BMI), alcohol use, smoking, income group, education and occupation. Those found to be significant at the 10% level were to be included in multivariate logistic regression to allow for the exploratory nature of the study. Multivariate logistic regression was subsequently used to explore associations of aTB with significant factors.

### 2.6 Ethics Statement

Participants provided written consent to participate in the survey and provided additional consent to undergo chest x‐rays. Clearance for the present study was provided by the University of Namibia Research Ethics Committee (Reference Number: SOM06/2023) and the Ministry of Health and Social Services (MoHSS).

## 3 Results

Overall, 29,495 participants in the survey underwent symptom screening, 27,751 had chest radiography and 7993 underwent sputum testing (Figure 1). The mean age of participants was 38 years (range 15–115) and median 34 years (interquartile range 23–48), with more females (16,547/29,495; 56.1%) than males (12,948/29,495; 43.9%).

**FIGURE 1:**
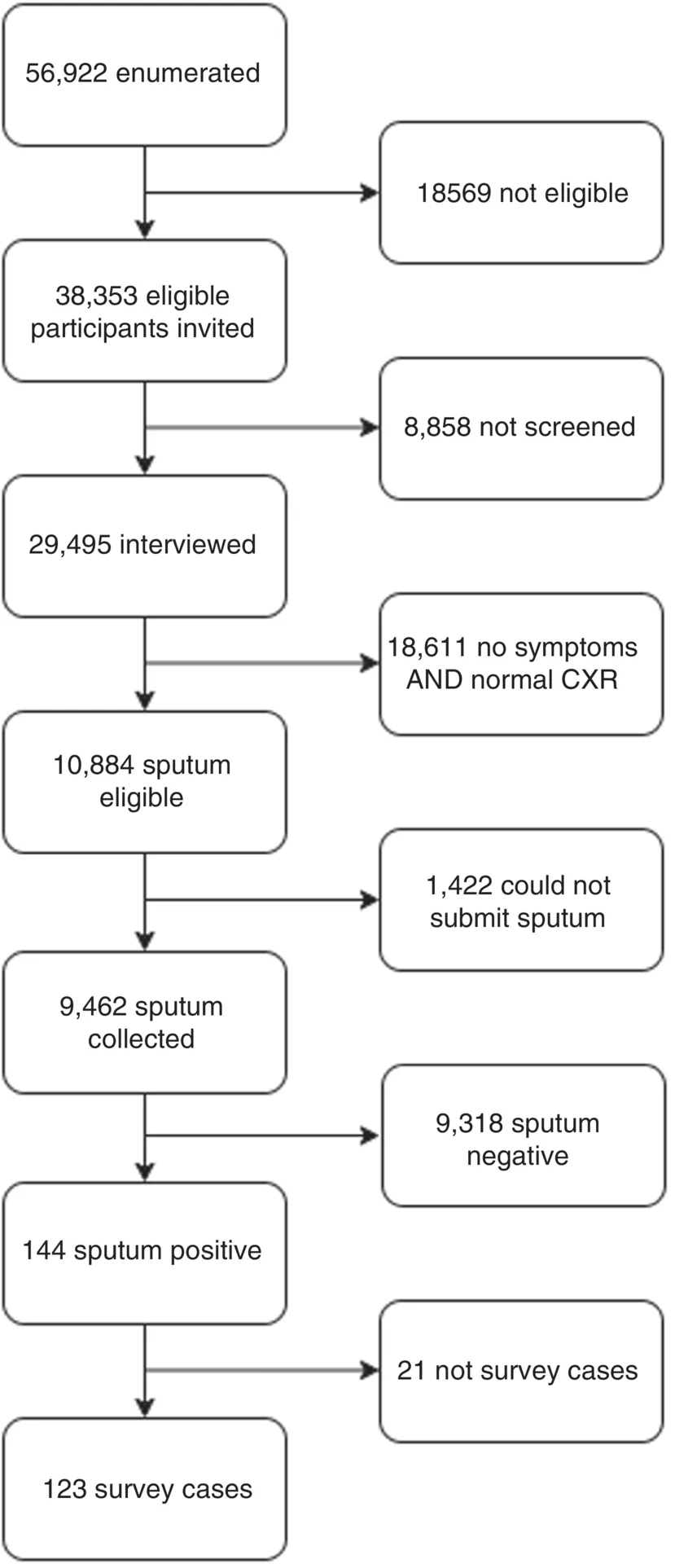
Flow of participants from the Namibia National Tuberculosis Prevalence Survey to the 123 bacteriologically confirmed tuberculosis cases included in this analysis.

### 3.1 Characteristics of TB Cases

There were 123 survey TB cases reported in total, with a median age of 44 years (interquartile range, 32–57 years). There were more males (74/123, 60.2%) than females (49/123, 39.8%) and 102/123 (82.9%) had an HIV result available, of whom 19/123 (18.6%) were HIV‐infected. Characteristics of participants are outlined in Table 1.

**TABLE 1:** Characteristics of the 123 bacteriologically confirmed tuberculosis cases in the Namibia National Tuberculosis Prevalence Survey, overall and among cases classified as asymptomatic under each of four case definitions.

Characteristic	Total (*N* = 123)	Definition 1 (no persistent cough)	Definition 2 (no cough)	Definition 3 (no W4SS)	Definition 4 (no persistent W4SS)
Cases, *N* (%)	123 (100%)	80 (65.0%)	63 (51.2%)	49 (39.8%)	63 (51.2%)
CAD4TB score (mean)	79.0	76.8	75.9 (*p* = 0.072)	77.3 (*p* = 0.429)	78.6 (*p* = 0.807)
Sex					
Female	49 (39.8%)	36 (45.0%) (0.11)	30 (47.6%) (0.07)	21 (42.9%) (0.58)	28 (44.4%) (0.29)
Male	74 (60.2%)	44 (55.0%)	33 (52.4%)	28 (57.1%)	35 (55.6%)
Age group					
15–24	15 (12.2%)	9 (11.2%) (0.47)	6 (9.5%) (0.44)	3 (6.1%) (0.33)	7 (11.1%) (0.57)
25–34	19 (15.4%)	10 (12.5%)	7 (11.1%)	6 (12.2%)	8 (12.7%)
35–44	29 (23.6%)	17 (21.2%)	14 (22.2%)	12 (24.5%)	13 (20.6%)
45–54	25 (20.3%)	19 (23.8%)	16 (25.4%)	12 (24.5%)	15 (23.8%)
55–64	17 (13.8%)	11 (13.8%)	9 (14.3%)	6 (12.2%)	8 (12.7%)
65+	18 (14.6%)	14 (17.5%)	11 (17.5%)	10 (20.4%)	12 (19.0%)
Geography					
Urban	55 (44.7%)	31 (38.8%) (0.07)	27 (42.9%) (0.67)	21 (42.9%) (0.74)	26 (41.3%) (0.43)
Rural	68 (55.3%)	49 (61.3%)	36 (57.1%)	28 (57.1%)	37 (58.7%)
HIV status					
Negative	83 (67.5%)	56 (70.0%) (0.66)	43 (68.3%) (0.93)	32 (65.3%) (0.92)	44 (69.8%) (0.85)
Positive	19 (15.4%)	12 (15.0%)	9 (14.3%)	8 (16.3%)	9 (14.3%)
Unknown	21 (17.1%)	12 (15.0%)	11 (17.5%)	9 (18.4%)	10 (15.9%)
Past TB treatment					
No	96 (78.0%)	66 (82.5%) (0.10)	53 (84.1%) (0.10)	42 (85.7%) (0.10)	53 (84.1%) (0.10)
Yes	27 (22.0%)	14 (17.5%)	10 (15.9%)	7 (14.3%)	10 (15.9%)
Household income					
Lowest	39 (34.2%)	23 (31.1%) (0.54)	18 (31.6%) (0.60)	15 (34.1%) (0.72)	19 (32.8%) (0.80)
Middle	38 (33.3%)	27 (36.5%)	18 (31.6%)	13 (29.5%)	21 (36.2%)
Highest	37 (32.5%)	24 (32.4%)	21 (36.8%)	16 (36.4%)	18 (31.0%)
Education level					
None	31 (25.6%)	23 (29.1%) (0.37)	16 (25.8%) (0.82)	13 (27.1%) (0.10)	19 (30.6%) (0.02)
Primary	46 (38.0%)	27 (34.2%)	22 (35.5%)	13 (27.1%)	16 (25.8%)
Secondary+	44 (36.4%)	29 (36.7%)	24 (38.7%)	22 (45.8%)	27 (43.5%)
Occupation					
None	80 (65.6%)	55 (69.6%) (0.31)	43 (69.4%) (0.67)	33 (68.8%) (0.78)	43 (69.4%) (0.67)
Unskilled	24 (19.7%)	15 (19.0%)	11 (17.7%)	8 (16.7%)	11 (17.7%)
Skilled/Prof	18 (14.8%)	9 (11.4%)	8 (12.9%)	7 (14.6%)	8 (12.9%)
Biomass (cooking)					
No	39 (31.7%)	27 (33.8%) (0.51)	21 (33.3%) (0.69)	15 (30.6%) (0.83)	21 (33.3%) (0.69)
Yes	84 (68.3%)	53 (66.2%)	42 (66.7%)	34 (69.4%)	42 (66.7%)
Biomass (heating)					
No	62 (50.4%)	39 (48.8%) (0.62)	31 (49.2%) (0.79)	25 (51.0%) (0.91)	32 (50.8%) (0.93)
Yes	61 (49.6%)	41 (51.2%)	32 (50.8%)	24 (49.0%)	31 (49.2%)
Smoking (active)					
No	76 (62.3%)	53 (67.1%) (0.14)	41 (66.1%) (0.37)	32 (66.7%) (0.42)	42 (67.7%) (0.21)
Yes	46 (37.7%)	26 (32.9%)	21 (33.9%)	16 (33.3%)	20 (32.3%)
Alcohol use					
No	65 (53.3%)	44 (55.7%) (0.47)	38 (61.3%) (0.07)	27 (56.2%) (0.60)	33 (53.2%) (0.99)
Yes	57 (46.7%)	35 (44.3%)	24 (38.7%)	21 (43.8%)	29 (46.8%)
Low BMI					
No	71 (59.7%)	50 (64.9%) (0.11)	41 (67.2%) (0.09)	34 (72.3%) (0.02)	39 (65.0%) (0.23)
Yes	48 (40.3%)	27 (35.1%)	20 (32.8%)	13 (27.7%)	21 (35.0%)

*Note:* Values are *n* (%) or mean unless stated; *p* values are from bivariate comparisons of asymptomatic versus symptomatic cases for each definition. Percentages are not weighted.

Abbreviations: aTB, asymptomatic tuberculosis; BMI, body mass index; CAD4TB, computer‐aided detection for tuberculosis; Definition 1, no persistent cough (≥ 2 weeks); Definition 2, no cough of any duration; Definition 3, no W4SS symptom of any duration; Definition 4, no W4SS symptom lasting ≥ 2 weeks; W4SS, WHO four‐symptom screen (cough, fever, weight loss, night sweats).

### 3.2 Presence of Symptoms in TB Cases

The most frequently reported symptom was any cough, presenting in 60 participants (48.8%), followed by any night sweats (21.1%) and any weight loss (20.3%). Fever (any duration) occurred in 20 TB patients (16.3%). Of the persistent symptoms (≥ 2 weeks duration), cough occurred in 43 (35.0%) patients, while night sweats and weight loss had an equal occurrence of 17.9%. Persistent fever was reported in 9.8% of patients (Table 2).

**TABLE 2:** Frequency of individual tuberculosis symptoms among the 123 confirmed survey cases.

Symptom	Frequency of any duration	Proportion any duration	Frequency ≥ 2 weeks	Proportion ≥ 2 weeks	*N*
Cough	60	48.8%	43	35.0%	123
Chest pain	37	30.3%	30	24.6%	122
Night sweats	26	21.1%	22	17.9%	123
Weight loss	25	20.3%	22	17.9%	123
Fever	20	16.3%	12	9.8%	123
Haemoptysis*	2	3.2%	2	3.2%	62*

*Note:* Values are *n* and percentages of the survey cases assessed; denominators vary slightly owing to missing responses.

* Only 62 participants were assessed for haemoptysis.

As shown in Figure 2, all four symptoms (any duration) were simultaneously reported in five (4.1%) patients and the least common symptom combination was fever with weight loss (one participant) as well as fever with night sweats (one participant). Cough alone presented in 28 participants (22.8%), while 49 participants (39.8%) had no symptoms at all. Figure 2 shows the relative prevalence of symptom combinations among survey TB cases.

**FIGURE 2:**
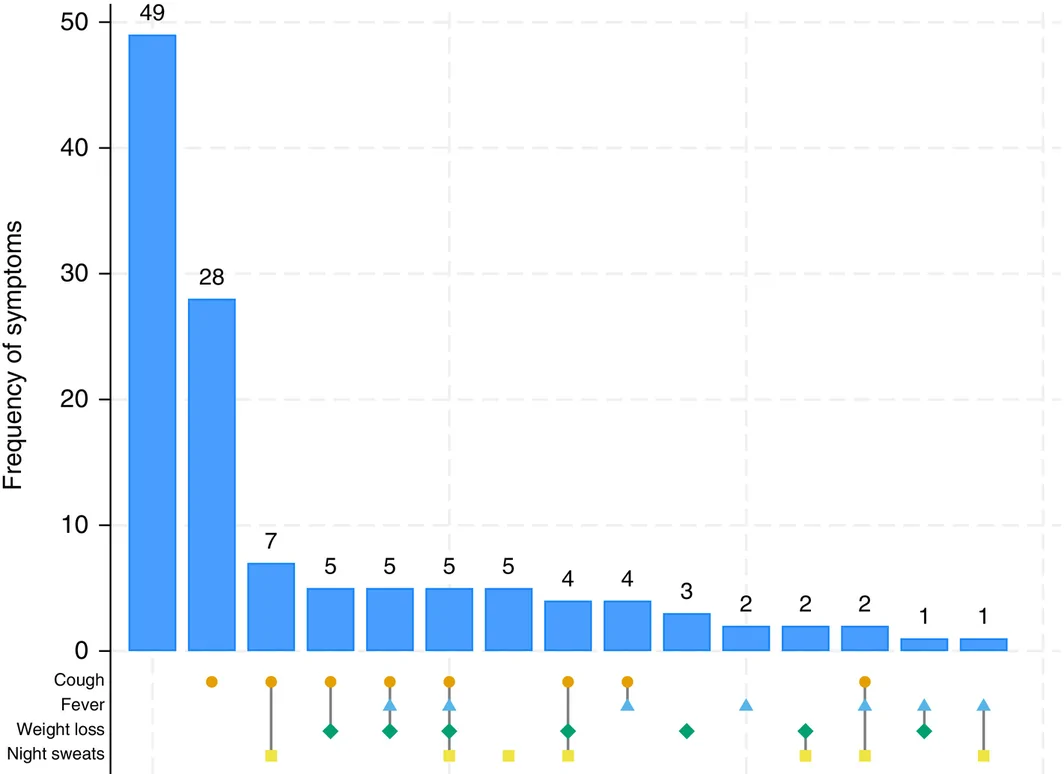
UpSet plot of tuberculosis symptom combinations (any duration) among the 123 confirmed survey cases (*N* = 123). The dot matrix indicates which symptoms make up each combination, and the vertical bars show the number of cases with that combination.

### 3.3 Proportion With Asymptomatic TB

The proportion of people with aTB among the survey cases varied by definition as shown in Figure 3 and Table 3, with Definition 1 (no persistent cough) being the most common (63.0%; 95% CI 55.3–71.8). Patients fulfilling Definition 2 (no cough of any duration) and Definition 4 (no W4SS of any duration) had almost similar proportions at 50.5% (95% CI; 41.2–59.8) and 50.1% (95% CI; 42.0–58.1), respectively. Those with Definition 3 (no persistent W4SS) had the lowest prevalence at 39.8% (95% CI; 31.1–49.1).

**FIGURE 3:**
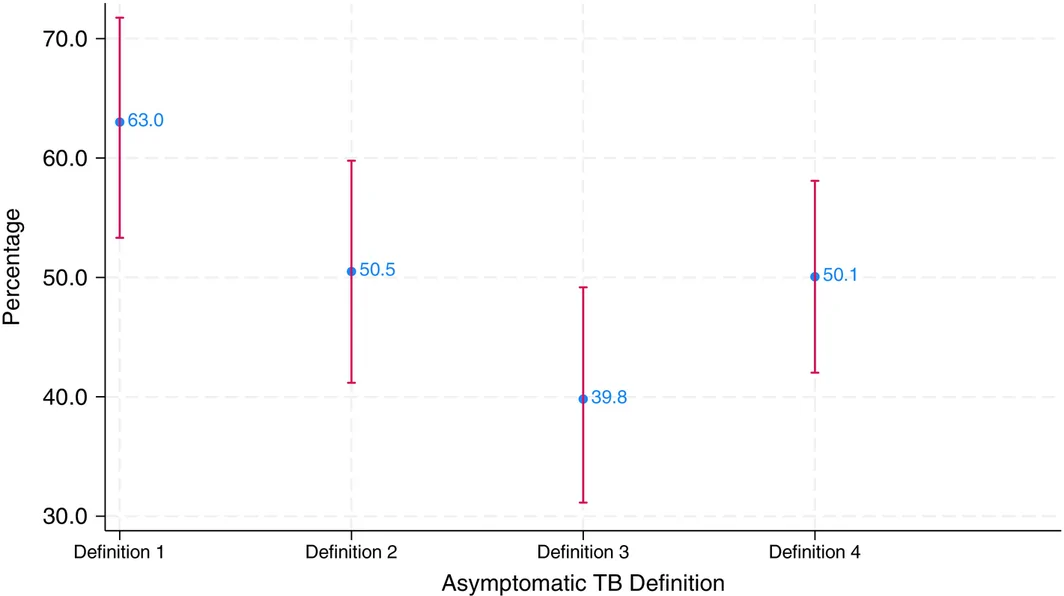
Survey‐weighted proportion of confirmed tuberculosis cases classified as asymptomatic under each of the four case definitions, with 95% confidence intervals. Definition 1, no persistent cough; Definition 2, no cough of any duration; Definition 3, no W4SS symptom of any duration; Definition 4, no W4SS symptom lasting ≥ 2 weeks.

**TABLE 3:** Weighted proportions of asymptomatic TB by definition (National TB Prevalence Survey, Namibia, subset *n* = 123*).

Definitions	Proportion	95% Confidence intervals	
Asymptomatic 1 (Persistent cough)	63.0%	55.3%	71.8%
Asymptomatic 2 (Any cough)	50.5%	41.2%	59.8%
Asymptomatic 3 (Any W4SS)	39.8%	31.1%	49.2%
Asymptomatic 4 (Any W4SS > 2 weeks)	50.1%	42.0%	58.1%

*Note:* Weighting accounts for the survey's complex sampling design, so these proportions differ from the unweighted counts in Table 1.

Abbreviations: CI, confidence interval; W4SS, WHO four‐symptom screen.

### 3.4 Risk Factors Associated With Asymptomatic TB

Age, sex, geography, CAD4TB score, HIV status, BMI, alcohol use, smoking, income, education and occupation were not significantly associated with aTB using Definition 1 at 5% significance. At the 10% significance level, a lower CAD4TB score (*p* = 0.081) and being in a rural area (*p* = 0.070) were associated with aTB.

Similarly, none of the aforementioned risk factors were associated with Definition 2 at the 5% significance level, but sex (*p* = 0.071), CAD4TB score (*p* = 0.072), past TB treatment (*p* = 0.095), alcohol use (*p* = 0.071) and BMI (*p* = 0.085) were associated at a 10% significance level. With Definition 3, BMI was a significant factor at 5% significance (*p* = 0.023) and past TB treatment at 10% (*p* = 0.095). With Definition 4, educational level had a significant association at 5% (*p* = 0.018), whereas past TB had a weaker association at 10% significance (*p* = 0.095).

In a multivariate logistic regression (Table 4) including all the significant factors from the bivariate analysis (sex, geography, alcohol use, BMI, past TB treatment, education), being in a rural area increased the odds of having aTB Definition 1 (AOR 2.62; 95% CI 1.03–6.63) while having a primary school education appeared to be protective against aTB Definition 4 compared to those with no schooling (AOR 0.28; 95% CI 0.08–1.00), an effect that was lost among those with secondary education and higher. No other factors showed a statistically significant association with aTB.

**TABLE 4:** Survey‐weighted logistic regression on factors associated with asymptomatic TB under each of the four definitions.

Predictor	Asymptomatic 1 OR (95% CI)	Asymptomatic 2 OR (95% CI)	Asymptomatic 3 OR (95% CI)	Asymptomatic 4 OR (95% CI)
Sex (Ref: Female)				
Male	0.42 (0.14–1.28)	0.62 (0.23–1.67)	0.88 (0.37–2.09)	0.50 (0.18–1.35)
Geography (Ref: Urban)				
Rural	2.62 (1.03–6.63)	1.39 (0.55–3.51)	1.68 (0.62–4.57)	1.94 (0.85–4.47)
Alcohol (Ref: No)				
Yes	0.67 (0.29–1.54)	0.40 (0.17–0.98)	0.58 (0.25–1.31)	0.93 (0.37–2.36)
Low BMI (Ref: No)				
Yes	0.68 (0.29–1.57)	0.59 (0.28–1.24)	0.49 (0.21–1.16)	0.76 (0.38–1.54)
Past TB (Ref: No)				
Yes	0.41 (0.15–1.13)	0.39 (0.14–1.11)	0.42 (0.12–1.40)	0.46 (0.17–1.27)
Education (Ref: None)				
Primary	0.41 (0.14–1.18)	0.68 (0.23–2.04)	0.45 (0.12–1.63)	0.28 (0.08–1.00)
Secondary & higher	0.68 (0.20–2.35)	0.67 (0.17–2.60)	0.99 (0.24–4.09)	1.04 (0.31–3.50)
CAD4TB (per unit)	1.00 (0.97–1.02)	1.00 (0.98–1.02)	1.01 (0.99–1.02)	1.01 (0.99–1.03)

*Note:* Models include the variables significant in bivariate analysis (sex, geographical area, alcohol use and body mass index). Values are odds ratios (95% confidence intervals); reference categories are indicated in the table.

Abbreviations: Asymptomatic 1, Asymptomatic TB definition 1—no persistent cough; Asymptomatic 2, Asymptomatic TB definition 2—no cough of any duration; Asymptomatic 3, Asymptomatic TB definition 3—no W4SS of any duration; Asymptomatic 4, Asymptomatic TB definition 4—no persistent W4SS; BMI, body mass index; CAD4TB, Computer aided detection for TB; CI, confidence interval; OR, odds ratio; W4SS, WHO four‐symptom screen.

## 4 Discussion

We found that in this cohort of Namibian patients diagnosed during a nationwide prevalence survey, the proportion of aTB varied according to the suite of symptoms used to define aTB. The proportion was highest with the use of cough for 2 weeks or more (63.0%) and lowest with the use of W4SS of any duration (39.8%). It is likely that, with such differences in the same group of patients as observed in our sample, inconsistencies will be observed when attempting to determine the prevalence of aTB in general populations. A scoping review by Teo et al. [14] confirmed the inconsistencies in the literature, with definitions ranging from aTB being completely asymptomatic to below a certain threshold of symptoms. In the accompanying meta‐analysis, studies reported a higher proportion of aTB using cough alone (any duration) compared to any other symptom combination [14].

We confirmed that the use of prolonged cough in symptom screening algorithms misses the highest number of TB cases, although our rates were higher than a similar study from Zambia [19], where prolonged cough missed 40%. After applying standardised definitions to individual participant data across 12 national prevalence surveys, Stuck et al. [18] noted that over 63% of people with pulmonary TB in community settings did not report cough at all, and 83% did not report persistent cough. In the same meta‐analysis, 28% of the patients did not report any typical symptoms as per W4SS, compared to our finding of 39.8% (95% CI 31.1%–49.1%). In the present study, we used the three definitions applied in Stuck's meta‐analysis and obtained comparable results. In many other studies, the requirement for persistent cough is widely used in clinical screening and misses over half of those with TB disease [14]. In our study, a definition that defines aTB according to persistent W4SS increases the proportion of aTB to 50.1%, although the increase is not statistically significant when compared with Definition 3 (any W4SS).

In our study, we only found statistically significant negative associations in the univariate analysis between BMI and aTB Definition 3 as well as between having a primary school education and aTB Definition 4. The former may be due to the inclusion of weight loss (any duration) in aTB Definition 3; patients with low BMI would likely have reported weight loss and those could not have aTB Definition 3. This association is lost in the multivariate analysis, suggesting a confounding effect. The fact that those in the rural areas were more likely to have asymptomatic TB if Definition 1 (lack of prolonged cough) was applied may likely be a function of health‐seeking behaviour and access to care. The protective effect of a primary school education against aTB 4 compared to no education suggests that participants with a primary school education were more likely to report symptoms as having lasted 2 weeks or more, possibly due to their literacy and reluctance to explain away symptoms. However, the loss of a statistically significant difference with those who completed secondary education or higher suggests an inconsistent effect or chance finding, possibly due to the small sample size and warrants further investigation in larger studies.

There is inconsistent evidence on how sociodemographic and other factors are associated with aTB compared to clinical TB. In the present study, this varied according to the suite of symptoms used in the definition. In community screening in neighbouring Zambia and South Africa, HIV and current smoking were found to be associated with aTB [22]. In an individual patient data meta‐analysis including 16 national surveys, smoking and diabetes were associated with symptomatic TB, but only smoking was associated with aTB, compared to no TB [23]. In the meta‐analysis by Stuck et al., being male was associated with a lower risk of aTB, but only for Definitions 1 and 2. Despite the concern that HIV may influence symptom presentation in TB patients [24], there has not been any consistent association with asymptomatic presentation in the meta‐analysis [2, 18, 23]. However, in a pooled estimate of studies on PLHIV, the proportion of patients with aTB was higher among those with higher CD4 counts (49%) than among those with low CD4 counts (22%) [14]. Additionally, HIV does seem to have an effect on the sensitivity of various symptom‐based screening algorithms, and better‐performing algorithms for symptom screening are still yet to be widely adopted. A prediction model developed by Yang et al. [25] was able to outperform the W4SS in PLHIV and “any TB symptom” as recommended by WHO in the HIV negative or unknown, in terms of sensitivity, while maintaining high specificity. This model, which proposes a scoring‐based algorithm depending on HIV status, was implemented in a high HIV and high TB setting in South Africa and Zambia [25].

The high prevalence of aTB in community settings should be a concern to TB programmes. Potential progression of disease, continued transmission and the need for effective treatment would be key discussion points for national programmes. There is indeed evidence that potentially half of undiagnosed cases have aTB [2] and that aTB is responsible for two thirds of TB transmission [7]. As demonstrated by Dinkele et al. [5], viable bacilli can be recovered from patients with TB just from tidal breathing even if they are not coughing. Nguyen et al. [8] demonstrated that in community settings, infection rates among household contacts did not differ between symptomatic and asymptomatic index cases. Similar findings were noted among prisoners in a controlled environment [26]. The high incidence and prevalence rate of TB in countries like Namibia may indeed be a result of continued community transmission by asymptomatic cases. National and subnational programmes have been focusing on finding and treating symptomatic cases, potentially ignoring and/or missing asymptomatic TB cases.

The recently published WHO definitions will assist the programmatic scale‐up of diagnosis and subsequent management of aTB [27]. However, they do not address the concern raised by Pierce [12] who asserts that “active TB to one clinician may be subclinical TB to another, and categorical heterogeneity remains”. The differing proportions of aTB cases provided by the respective definitions in this study demonstrate this point. It is thus imperative that country TB programmes clarify which definition of aTB is being applied to facilitate accurate comparison across geographical locations and time [14]. This study adds to the evidence by applying four definitions of aTB in a high HIV prevalence setting.

The study limitations include the use of secondary data from a prevalence survey and the relatively small sample size. However, the approach used offers the most representative national sample in settings that have been demonstrated to detect aTB. Additionally, the data were not longitudinal; hence, progression and outcomes of aTB were not available for analysis. Linkage to the TB treatment register in future might give an indication of whether aTB cases were initiated on treatment.

Symptom‐negative radiologically negative cases were excluded because only those with symptoms or abnormal CXRs would have had sputum sent for testing and diagnosis. Additionally, this study did not analyse bacteriologically unconfirmed (clinically diagnosed) aTB as the criteria for diagnosing these were not available at the time of the survey. It might, therefore, be that fewer aTB cases were included in this study than actually existed in the pool of participants in the primary prevalence survey.

## 5 Conclusion

This study presents local evidence of the high proportion of patients with aTB within community settings in Namibia, which should be a concern for TB control efforts. There is a need to reduce reliance on symptoms alone for TB screening and diagnosis. We recommend the parallel use of symptom screening and CXR in any screening algorithm and the option to consider universal testing without screening.

## Funding

The authors have nothing to report.

## Conflicts of Interest

The authors declare no conflicts of interest.

## Data Availability

The data that support the findings of this study are available from the corresponding author upon reasonable request.
